# Growth and development of an invasive forest insect under current and future projected temperature regimes

**DOI:** 10.1002/ece3.9017

**Published:** 2022-06-17

**Authors:** Jonathan A. Walter, Lily M. Thompson, Sean D. Powers, Dylan Parry, Salvatore J. Agosta, Kristine L. Grayson

**Affiliations:** ^1^ Department of Biology University of Richmond Richmond Virginia USA; ^2^ Department of Environmental Sciences University of Virginia Charlottesville Virginia USA; ^3^ Department of Forestry and Environmental Conservation Clemson University Clemson South Carolina USA; ^4^ Integrative Life Sciences Doctoral Program Virginia Commonwealth University Richmond Virginia USA; ^5^ Department of Environmental Biology SUNY College of Environmental Science and Forestry Syracuse New York USA; ^6^ Center for Environmental Studies Virginia Commonwealth University Richmond Virginia USA

**Keywords:** climate warming, growth chamber, local adaptation, *Lymantria dispar*, physiology, reciprocal transplant

## Abstract

Temperature and its impact on fitness are fundamental for understanding range shifts and population dynamics under climate change. Geographic climate heterogeneity, behavioral and physiological plasticity, and thermal adaptation to local climates make predicting the responses of species to climate change complex. Using larvae from seven geographically distinct wild populations in the eastern United States of the non‐native forest pest *Lymantria dispar dispar* (L.), we conducted a simulated reciprocal transplant experiment in environmental chambers using six custom temperature regimes representing contemporary conditions near the southern and northern extremes of the US invasion front and projections under two climate change scenarios for the year 2050. Larval growth and development rates increased with climate warming compared with current thermal regimes and tended to be greater for individuals originally sourced from southern rather than northern populations. Although increases in growth and development rates with warming varied somewhat by region of the source population, there was not strong evidence of local adaptation, southern populations tended to outperform those from northern populations in all thermal regimes. Our study demonstrates the utility of simulating thermal regimes under climate change in environmental chambers and emphasizes how the impacts from future increases in temperature can vary based on geographic differences in climate‐related performance among populations.

## Graphical abstract

We combine precision environmental chambers with geographically explicit projections of future temperatures to test the larval performance under climate change of *Lymantria dispar* from several range‐edge populations. *L. dispar* is a highly damaging non‐native forest pest. Our findings suggest that future fitness increases that depend on geography and greenhouse gas emissions scenario and offer some evidence for local adaptation in thermal performance traits across the range of this non‐native species.

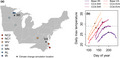

## 1 INTRODUCTION

Climate change is altering the geographic ranges and population dynamics of organisms across the globe (Parmesan & Yohe, 2003; Thomas, 2010). These changes reflect the accumulated effects of a shifting climate on individual fitness. Insects and other ectothermic taxa are thought to be especially susceptible to direct effects of climate change due to the temperature‐dependence of their vital rates (Björkman et al., 2011; Boggs, 2016); however, whether the net effects are positive or negative for population growth and viability can depend on contexts including geography, species life history, and how climate change impacts species interactions (Klapwijk et al., 2013; Van Dyck et al., 2015; Walter et al., 2018).

Many studies have quantified the physiological performance of various insect species in response to temperature (e.g., Fischer et al., 2011; Kingsolver & Woods, 1997), and postulated the consequences of generally increasing environmental temperatures on organismal survival and growth rates in the future. However, the ability to empirically test these responses is limited by the constraints and venues for experimental studies (Lindroth & Raffa, 2017). Highly controlled and replicated systems, such as growth chambers, can quantify thermal limits and reaction norms more easily across a range of conditions but often employ simplified representations of environmental conditions. Field venues, on the other hand, present logistical challenges for experimental replication and are subject to stochastic environmental changes unrelated to the experimental design (Pelini et al., 2011; Rich et al., 2015). For either venue, experimental studies of insect responses to climate warming often increase temperature in the lab or field by a constant (e.g., +1.7 or +3.0°C; Bauerfeind & Fischer, 2014; MacLean et al., 2017; Rich et al., 2015). Given that the effects of climate change on temperature differ geographically (Karmalkar & Bradley, 2017), seasonally (Kirk et al., 2019), and diurnally (Braganza et al., 2004), such studies may provide an incomplete view of the response of insects to future environmental temperatures under climate change.

These shortcomings can be overcome through the combination of modern environmental chambers and spatially downscaled climate projections that include greenhouse gas forcing scenarios adopted by the Intergovernmental Panel on Climate Change (IPCC), which have relatively recently become publicly available (Eyring et al., 2016; Taylor et al., 2012). When coupled with environmental chambers capable of fine‐scale temperature programming, these projections can be used to experimentally produce temperature regimes that more accurately reflect the temperature regimes found in nature. While other environmental factors that potentially contribute to changes in development and fitness (e.g., light, nutrient resources, water availability, air flow) are controlled in this type of growth chamber simulation, this allows for the effect of future temperature regimes on development and fitness to be evaluated independently. Despite its potential, however, the use of realistic temperature regimes in growth chamber has been underutilized (but see Bradshaw et al., 2004; Maguire et al., 2015; Sheffer et al., 2021; Williams et al., 2015).

Evaluating the performance of an organism under future climate change is complicated by the plastic and evolved responses to temperature that can occur across the geographic range of a species. Few empirical studies have examined how these differences could manifest under novel climates in the future (Sgrò et al., 2016; Yang et al., 2021). These geographic gradients in thermal performance can also develop during the range expansion of invasive species when encountering potentially wide‐ranging and novel climates over short timescales (Batz et al., 2020; Colautti & Lau, 2015; Kosmala et al., 2018). The gradual expansion of *Lymantria dispar dispar* (L.) (Lepidoptera: Erebidae) in Eastern North America over the past 150 years, is a well‐studied example of a system where these geographic gradients in thermal performance have developed across an invasion front. This species (common name ‘spongy moth’) is a forest‐defoliating generalist pest in North America that feeds on over 300 tree species (Liebhold et al., 1995). Its introduced range currently spans Canada and Minnesota to North Carolina, and its spread over the past ca. 150 years has been the subject of intensive study and detailed population monitoring at the invasion front for over two decades (Grayson & Johnson, 2018).

Previous studies have quantified the genetic basis for selection on temperature‐dependent development in *L. dispar* and the resulting local adaptation of ecologically important traits across the invasion (Faske et al., 2019; Friedline et al., 2019; Thompson et al., 2017, 2021). The detailed knowledge on the spread and thermal performance of this invasive species makes it an ideal organism to investigate thermal performance and fitness under realistic future temperature scenarios. Given the differences between populations seen in earlier studies, it is unclear how the effects of local adaptation will manifest in responses to future climate warming. To address this, we conducted an experimental study to address the following questions: (1) how does *L. dispar* growth and development respond to current climates across its invasive range; (2) how does *L. dispar* growth and development respond to projected 2080 climatic conditions; and (3) does this response vary among populations from different parts of its invasive range? We used growth chambers to simulate present‐day and projected future temperature regimes near the southern and northern extremes of the US invasion and measured growth and development in individuals from populations originating at the northern and southern range extremes. We hypothesized that climate warming would enhance fitness‐related traits relative to a contemporary climate baseline under northern range‐edge thermal conditions, but would reduce performance under southern range‐edge conditions. Moreover, we hypothesized that the magnitude of these effects will depend on source population due to a history of local adaptation to climate, with individuals from cooler climates tending to perform better in cooler temperature regimes and individuals from warmer climates being more tolerant of warming.

## 2 METHODS

### 2.1 Study system

Defoliation and tree damage from *L. dispar* (L.) (Lepidoptera: Erebidae) causes an average of $250 M USD of economic damage in the US annually (Aukema et al., 2011). Outbreaks of *L. dispar* have been implicated as a contributing factor to the decline of oaks (*Quercus* spp.) in eastern North America (Morin & Liebhold, 2016), and defoliation events alter ecosystem processes (Clark et al., 2010; Riscassi & Scanlon, 2009). Since its introduction to North America in the Boston, MA area in 1868 or 1869, *L. dispar* has expanded across a wide climatic gradient. Its northern invasive range limit, currently in Minnesota and Canada, is bounded by lethal cold temperatures for overwintering eggs and insufficient warmth to complete larval development within the shortened growing season (Gray, 2004; Streifel et al., 2019). The current southern range limit, currently in eastern Virginia and North Carolina, may be governed by supraoptimal summer temperatures and sublethal effects on growth, fecundity, and hatching success (Gray, 2004; Tobin et al., 2014).

Across the climatic gradient of the historic spread and current invasion front, several studies have found that *L. dispar* has experienced adaptation to local climates. For example, egg masses sourced from warmer climates had higher viability when reared at warm range‐edge temperatures relative to populations from cooler regions (Faske et al., 2019). In constant temperature experiments, warmer climate populations had lower mortality rates and smaller reductions in fitness‐associated traits when reared at supraoptimal temperatures than those sourced from cooler climates (Thompson et al., 2017, 2021). Additionally, genomic evidence is consistent with a genetic basis for phenotypic differences in temperature‐related performance traits (Friedline et al., 2019).

### 2.2 Experimental design

**FIGURE 1:**
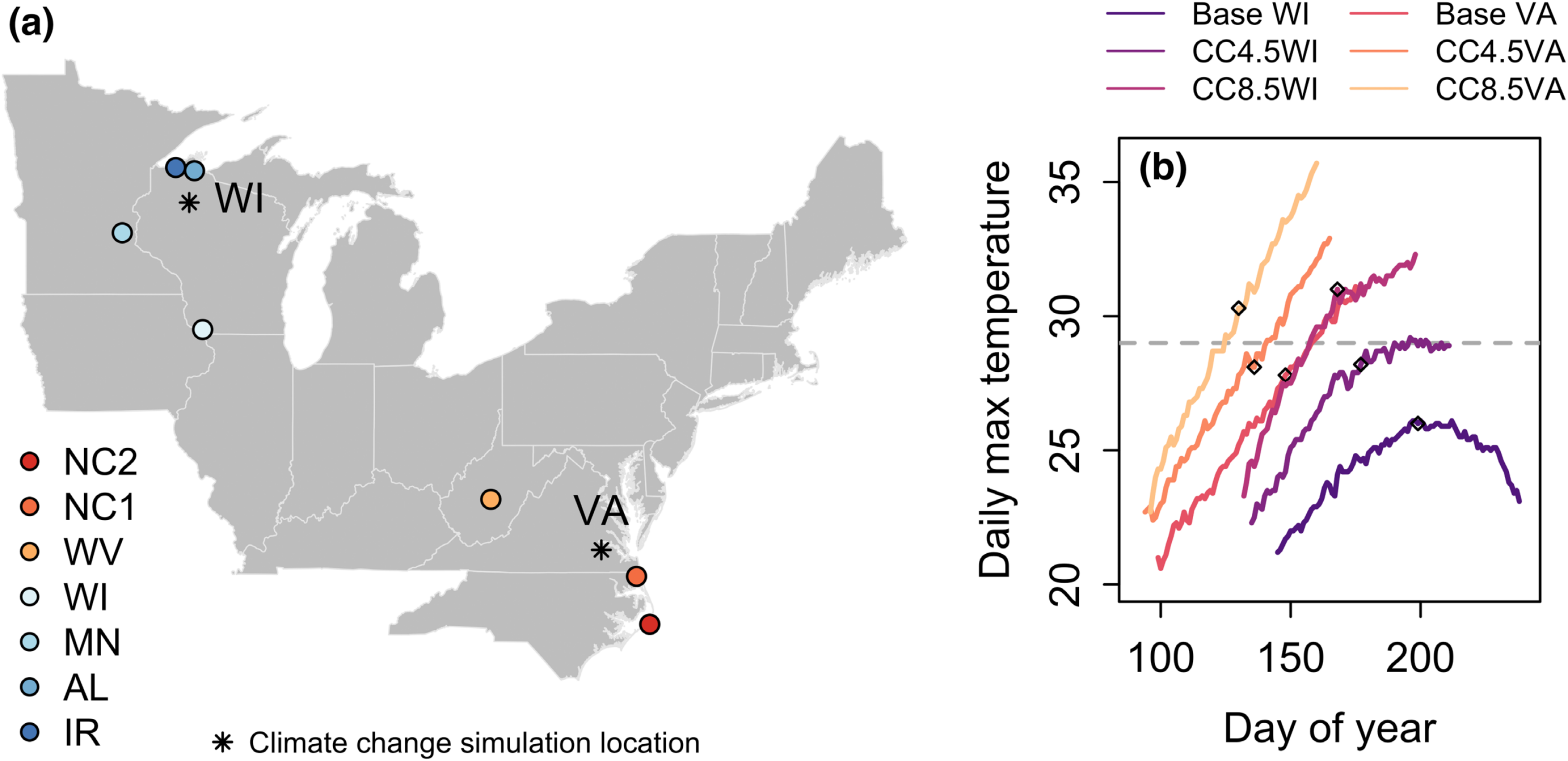
(a) Map of *L. dispar* source populations. The order from south to north also corresponds to the order of mean annual temperature at each source population location; (b) daily maximum temperature over time for simulated temperature treatments, for the time period spanning modeled egg hatch through adult emergence. The gray horizontal line indicates the across‐populations average thermal optimum for *L. dispar* larval development (29°C). Diamonds indicate empirical 95th percentile fifth instar maturation dates from this experiment

Individuals used in this experiment were sourced from seven populations in the Eastern US, a subset of those used in Thompson et al. (2021). These populations represent two regions, denoted North and South, which are areas of active range expansion and the current climatic extremes of the *L. dispar* invasive range in the US (Figure 1a). Each of the populations were collected from areas at the invasion front with low population density and we collected multiple separate populations from each region to serve as replicates. Eleven to 60 egg masses were collected from each source population, and egg masses from the same source population were homogenized and reared for at least one generation under consistent dietary and temperature conditions to control the potential site and maternal effects. Matings were haphazard, and egg masses from the same source population were homogenized between generations. Additional details on population sources can be found in Table S1. Individuals used in this experiment were transported and housed under USDA APHIS permits P526P‐17‐03681 (KLG) and P526P‐16‐04388 (DP).

Twenty‐five individuals from each source population were randomly selected from homogenized eggs from the source colonies and reared from egg hatch to adulthood (or mortality) in each of six thermal regimes representing present‐day temperatures from the North and South regions of the *L. dispar* invasion front and projected temperatures under climate change scenarios for 2080: Baseline WI, CC4.5WI, CC8.5WI, Baseline VA, CC4.5VA, and CC8.5VA (Figure 1b). The WI (North) and VA (South) designations correspond to locations in Wisconsin (WI; 45.7992°N, 90.9947°W) and Virginia (VA; 37.1122°N, 77.2017°W) for which we generated thermal regimes (Figure 1a). These locations are near the inland northwestern and southeastern extremes of the invasion front and represent extremes of cold and warmth experienced by *L. dispar* in the USA. The CC4.5 and CC8.5 designations correspond to moderate and more severe representative concentration pathways, scenarios for future greenhouse gas emissions that are used to drive climate projections.

The six temperature treatments differ in their mean temperatures (Baseline WI mean ± standard deviation = 16.6 ± 2.5; CC4.5 WI = 17.9 ± 5.0; CC8.5 WI = 22.9 ± 2.1; Baseline VA = 21.9 ± 4.2; CC4.5 VA = 24.2 ± 4.6; CC8.5 VA = 26.8 ± 4.9), the rate of increase in temperature through time (Figure 1b), and the degree to which temperatures may plateau and even decline before development to adulthood was projected to complete (Figure 1b). Projected climate change (respectively, RCP4.5 and RCP 8.5) increased mean daily maximum temperatures during development relative to their historical baselines by 1.78°C and 4.38°C in Virginia and by 2.51°C and 4.87°C in Wisconsin. Mean daily minimum temperatures increased by 2.07°C and 4.54°C in Virginia and by 2.58°C and 4.70°C in Wisconsin.

Temperature profiles were generated using BioSIM 10 software (Regniere & Saint‐Amant, 2017) and represent the mean of 200 replicate stochastic simulations of daily minimum and maximum temperatures generated from historical and projected future monthly climate normals (Régnière & St‐Amant, 2007). Baseline temperature regimes were based on 1981–2010 climate normals, and climate change scenarios were based on the CanRCM4 climate model ensemble (Scinocca et al., 2016). BioSIM statistically interpolates between nearby weather stations to estimate weather conditions at specific geographic locations. Simulated future temperature regimes for each of the four nearest weather stations were constructed by adjusting 1981–2010 monthly normals with temperature anomalies from CanRCM4 ensemble projections and interpolated to the specified locations using BioSIM. We aligned the start of each chamber temperature regime to the predicted date of median egg hatch as estimated using the *L. dispar* phenology model in BioSIM (Gray, 2004; Gray et al., 2001; Regniere & Sharov, 1997). The experiment began by introducing newly hatched larvae at the predicted hatch date for each simulation.

Temperature regimes were implemented in environmental chambers (Percival Scientific, Inc. model I‐22VL running Intellus Connect Ultra software) on a ramp between a daily minimum temperature at 7 am and a daily maximum temperature at 9 pm. The chambers maintained a 14‐h light, 10‐h dark cycle with lights on between 7 am and 9 pm. Humidity was monitored by electronic HumiChip sensors within the chambers and was maintained by water pans or desiccant to remain between 60% and 80% RH (relative humidity). The positions of individuals within the chamber were rotated to prevent microscale differences in air flow and light from having persistent effects on development.

Three temperature regimes each were housed in labs at Virginia Commonwealth University (Baseline VA, Baseline WI, CC4.5WI) and at University of Richmond (CC4.5VA, CC8.5VA, CC8.5WI). Both labs used the same model of growth chamber with all settings in common. Prior to the experiment, each environmental chamber was carefully calibrated for both light and dark cycles using two different ca. three‐day programs designed to ensure that each chamber maintained programmed temperatures within a tolerance of ±0.5°C. In the first calibration test, temperature stepped from 6°C to 16°C to 26°C. In the second, chambers were brought to a constant temperature over 8 h before implementing two daily cycles between low and high temperatures corresponding to the beginning of the VA base temperature regime. Temperature data loggers (HOBO U23 Pro v2, Onset Computer Corporation) were also placed inside each environmental chamber to track rearing temperatures across the experiment. These records showed that actual chamber temperatures tracked programmed temperatures across treatments, with only minor deviations that did not obscure differences among treatments (Figure S1). In addition, an earlier experiment conducted in these environmental chambers replicated two different fluctuating temperature thermal regimes in two chambers each, and found no differences in development times, masses, or survivorship between chambers implementing the same treatments (K. Grayson, *unpublished data*).

Larvae were housed in individual plastic cups with cubes of artificial diet (USDA APHIS formulation) that were replaced weekly. Individuals were checked daily between 10 am and 2 pm for changes in developmental stage. We recorded the following: third instar date, third instar mass, fifth instar date, fifth instar mass, pupation date, pupal mass, adult emergence date, and sex. Male *L. dispar* typically complete five developmental instars before pupation, while females typically complete six instars; thus the fifth instar is the last developmental stage where both sexes can be measured as larvae before pupation. The sex of individuals could not be determined if they died prior to the onset of sexual dimorphism that develops in late‐stage larvae. We focus here on data from third and fifth instars because fungal contamination of the artificial diet increased mortality between fifth instar and adulthood in individuals raised in one of the laboratories (Figure S2). Despite this, sample sizes were robust (92% of larvae survived to fifth instar); survivorship to fifth instar by source population and thermal regime is reported in Table S2. Data from this and a previous experiment (Thompson et al., 2021) showed that larval masses were strongly correlated with pupal masses (Table S3), which in turn, are strongly related to fecundity (Faske et al., 2019; Honěk, 1993).

### 2.3 Analyses

We analyzed how growth rate and development time from hatch to third and to fifth larval instars depended on temperature regime (Baseline WI, Baseline VA, CC4.5 WI, CC4.5 VA, CC8.5 WI, CC8.5 VA), region (South, North), and the interaction between temperature regime and region using linear mixed‐effects models. Growth rates were computed as the difference from neonate mass divided by the development time, expressed in g day^−1^. Because individual neonate masses were below the precision of standard analytical balances, we instead obtained a starting larval mass for each population by weighing five replicate groups of five neonate larvae from each source population and taking the average. Although the use of relative growth rates can sometimes improve inferences by correcting for differences in starting mass, neonate masses did not statistically differ by source population. Temperature regime and region were each coded as categorical variables. We included in our statistical models a fixed effect of sex coded as a numeric variable with male = −1, unknown = 0, and female = 1 to account for *L. dispar* becoming sexually dimorphic later in development. Individuals of unknown sex did not survive long enough to visually determine sex and were coded an intermediate value. We expected a 50:50 sex ratio, so the unknown group very likely includes males and females. We also included a random effect of source population on the intercept. Given that the experiments were conducted within high‐performance environmental chambers housed within modern climate‐controlled laboratories in close geographical proximity, it is unlikely that differences between laboratories in chamber operation influenced our results, but we cannot rule it out. Analyses were conducted using linear mixed‐effects models with the ‘lmerTest’ package (Kuznetsova et al., 2017) in R version 3.6.1 (R Core Team, 2020). Significance of model terms was assessed using Wald *Χ* ^2^ tests with type‐III sums of squares. Statistical significance of comparisons between groups was determined post hoc based on 95% confidence intervals of estimated marginal means. In addition, we used the ‘effectsize’ R package (Ben‐Shachar et al., 2020) to compute partial *η* ^2^ for the fixed effects in our statistical models, which corresponds to the proportion of the total variance in the response variable that can be statistically attributed to each term, after accounting for other terms in the model.

## 3 RESULTS

**FIGURE 2:**
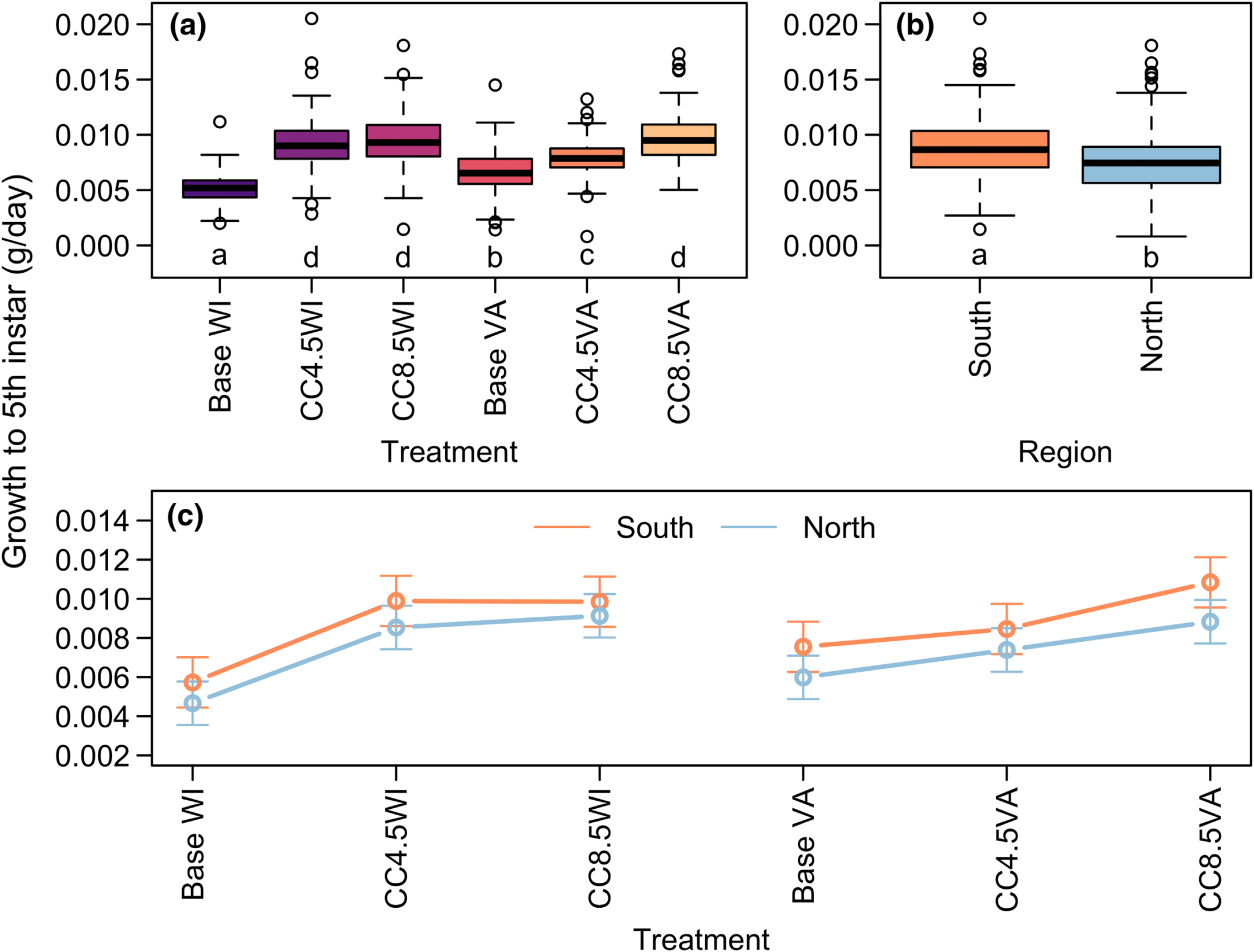
Fifth instar *L. dispar* larval growth rates by (a) thermal regime treatment, (b) region, and (c) region‐by‐thermal regime treatment interaction for all individuals. Lowercase letters in panels (a) and (b) denote groups whose elements have estimated marginal means with overlapping 95% confidence intervals. Error bars in (c) indicate 95% confidence intervals. Sex was accounted for in statistical models as a fixed effect but was not of primary interest for this study

The effects of temperature regime, region, temperature regime‐by‐region interaction, and sex on larval growth rates were consistent for growth to third and to fifth instars. We focus on results for fifth instars; parallel results for third instars are shown in Figures S3 and S4. Growth rates of fifth instar larvae differed by temperature regime (df = 5, *Χ* ^2^ = 852.3, *p* < .0001, *η* ^2^ = 0.47), region (df = 1, *Χ* ^2^ = 3.85, *p* = .0498, *η* ^2^ = 0.44), and sex (df = 1, *X* ^2^ = 11.50, *p* < .0001, *η* ^2^ = 0.01), with a statistically significant two‐way interaction between treatment and region (df = 5, *Χ* ^2^ = 13.84, *p* = .017, *η* ^2^ = 0.01). Growth rates to fifth instar tended to increase in thermal regimes corresponding to climate change projections (Figure 2a), and were highest in the CC4.5WI, CC8.5WI, and CC8.5VA treatments. Larvae from Northern source populations tended to gain mass more slowly than those from Southern source populations (Figure 2b). The thermal regime‐by‐region interaction effect suggests that *L. dispar* larvae have some tendency to respond to climate warming by increasing growth rate more so in simulated future climates that represented the region from where they were sourced, although this effect was weak (Figure 2c). The clearest example of this can be seen in comparing region‐specific mean growth rates between the CC4.5 VA and the CC8.5VA temperature regimes (Figure 2c), where the rate of mass gain for individuals from southern populations increased more strongly between the two temperature regimes. Note that the CC8.5VA regime had the warmest temperatures (Figure 1b).

**FIGURE 3:**
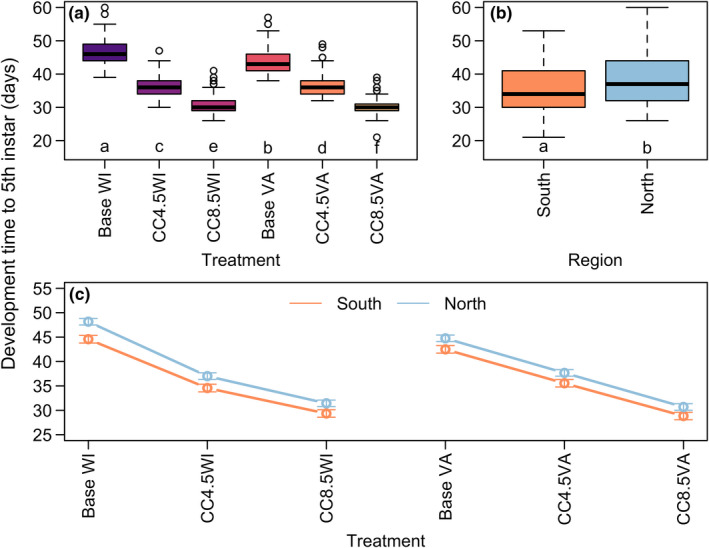
Development time of *L. dispar* to fifth instar by (a) thermal regime treatment, (b) region, and (c) region‐by‐thermal regime treatment interaction for all individuals. Lowercase letters in panels (a) and (b) denote groups whose elements have estimated marginal means with overlapping 95% confidence intervals. Error bars in (c) indicate 95% confidence intervals. Sex was accounted for in statistical models as a fixed effect but was not of primary interest for this study

The general pattern of effects of temperature regime, region, and temperature regime‐by‐region interaction on development time to fifth instar was consistent with patterns observed for growth rates despite the correlation between growth rate and development time (Pearson correlation = −0.69) accounting for <50% of variation between them. Across thermal regimes, development times decreased from cooler to warmer treatments (Figure 3a; df = 5, *X* ^2^ = 5230, *p* < .0001, *η* ^2^ = 0.85), and development times tended to be longer for northern populations than southern (Figure 3b; df = 1, *X* ^2^ = 54.89, *p* < .0001, *η* ^2^ = 0.92). Despite a marginally statistically significant interaction effect between temperature regime and region (df = 5, *X* ^2^ = 10.69, *p* = .058, *η* ^2^ = 0.01), there were minimal qualitative effects of this statistical interaction (Figure 3c). There was also a statistically significant effect of sex in which females tended to grow more slowly than males (df = 1, *X* ^2^ = 166.0, *p* < .0001, *η* ^2^ = 0.15).

## 4 DISCUSSION

Our study examined how growth and development of an invasive insect manifests under contemporary and future thermal regimes using populations of *L. dispar* sourced from the current climatic extremes of the introduced range. We used thermal regimes that simulated future climate warming and found increases in larval growth and development rates. As hypothesized, warming increased growth rates and reduced development times at the northern range edge, but contrary to expectations, warming did not make conditions at the southern range boundary hot enough to result in sublethal impacts to larval growth and development. Taking larval growth and development as an index of fitness based on the correlation between mass and fecundity (Faske et al., 2019) and the reduction in exposure to natural enemies from more rapid development, our findings suggest that future changes in fitness will depend on the region and magnitude of temperature change, assuming equivalence of host plant resources. If climate warming causes geographically dependent increases in *L. dispar* fitness, there would be substantial effects on the future ecological and economic impacts of this forest pest, and on allocation of management efforts to slow its spread.

We found evidence that source populations responded somewhat differently to experimental thermal regimes (Figures 2c and 3c). However, evidence for local adaptation of larval growth and development was equivocal overall, in contrast to other recent studies (Faske et al., 2019; Friedline et al., 2019; Thompson et al., 2017, 2021). Regional variation in larval growth rates in the baseline and CC4.5 temperature regimes was not discernibly related to a “home field advantage,” although the regions diverged more strongly in the CC8.5 temperature regimes. One possibility is that only in the CC8.5 regimes did supraoptimal temperatures occur frequently enough during larval development for growth rates to diverge. Because this experiment used fluctuating temperatures, periods of supraoptimal temperatures were transient, even under our climate change scenarios. There is evidence that southern populations are more tolerant of high temperatures (Thompson et al., 2017, 2021), which could explain their better performance in our hottest treatments. The higher overall growth rate in southern populations is consistent with the converse‐Bergmann's rule explaining geographic gradients in ectotherm body size where larger body sizes are found in lower latitude populations with warmer climates (Shelomi, 2012). However, we found no clear evidence that northern populations can perform better than southern populations in cooler temperature regimes (Figures 2c and 3c). Overall, these results continue to demonstrate that while larval growth and development in *L. dispar* has undergone climate‐related adaptation, these traits remain plastic in response to temperature (Thompson et al., 2021).

One caveat to our study is that the scope of our analysis is limited to larval life stages. A mold outbreak increased mortality between the fifth instar, pupal, and adult life stages in some treatments (Baseline VA, Baseline WI, CC4.5 WI) and limited the scope of our analyses to larval life stages before the initiation of pupation. While sample sizes of surviving individuals permitted robust statistical analysis, we cannot fully rule out that there were sub‐lethal or pre‐lethal effects on the growth and development of larvae in affected treatments. The most likely impact of any unknown mold effects on our findings is to accentuate differences between cooler and warmer temperature regimes by depressing growth rates or increasing development times in cooler temperature regimes. There is no a priori reason to expect that our source populations differ in their resistance to mold, and our practice of rotating the locations of individuals to prevent artifacts of location within the growth chamber likely randomized spread of mold. Therefore, we think it unlikely that mold affected our conclusions about local adaptation, and because the temperature regimes with greatest warming were unaffected by mold, it has no bearing on our conclusion that *L. dispar dispar* larvae grew better than expected in the treatments representing warmer climates.

The potential responses of insects and other ectotherms to future climate change have largely been evaluated using predictions modeled from species‐specific thermal performance curves (Kingsolver et al., 2013; Sinclair et al., 2016; von Schmalensee et al., 2021) or studies that experimentally elevate temperature in a lab or field setting. Here, we instead used an innovative approach allowing us to make realistic inferences about organismal performance under climate warming scenarios. As climate change has non‐uniform effects on the spatiotemporal distributions of temperatures (Braganza et al., 2004; Karmalkar & Bradley, 2017; Kirk et al., 2019), our approach represents a substantial advance in methodology compared with simply increasing temperature by a constant value from a current baseline. Indeed, the thermal regimes that we derived from climate change projections differed not only in their means but also in the rate of increase in temperature through time (Figure 1b).

These benefits of being able to program projected thermal regimes in detail are weighed against the simplistic growing environment in chambers, which often lack community interactions with other species, compared with open‐air warming experiments in the field, which permit these complexities, but are also logistically challenging and resource‐intensive (Rich et al., 2015). Notably, controlled chamber settings focus the mechanistic inference on the effects of temperature rather than the complex interactions from other environmental variables or interactions. Programmed environmental chambers provide the additional benefit of being able to test realistic thermal regimes from any geographic position or point in time independent of physical location. While some laboratory studies have simulated detailed and realistic thermal regimes (Bradshaw et al., 2004; Maguire et al., 2015; Sheffer et al., 2021; Williams et al., 2015), we are unaware of other studies using growth chambers to simulate different, realistic, climate change scenarios for multiple locations. This is likely in part because its feasibility depends on modern environmental chamber control software and a study system with some a priori knowledge of phenology, but we encourage this design as a means of increasing the realism of climate change ecophysiology studies in controlled experimental settings. As wild organisms experience and respond to microclimatic variation in ways that organisms generally cannot in an experimental setting, it is important to note that experimental designs like ours are best suited for capturing larger scale spatial variation in temperatures.

Future *L. dispar* performance with climate warming could make range expansion and population outbreaks more common in the northern range extremes. Indeed, establishment of populations in northern Minnesota has occurred in areas earlier predicted to be marginal for survival (Streifel et al., 2019). Conversely, at the southern extreme, range stasis and retraction has already been observed (Tobin et al., 2014) but our results suggest that larval development at the southern range edge is not impaired by present or projected future temperature regimes. Reduced egg viability, possibly due to insufficient cold to complete diapause, could be the mechanism for range stasis at the southern extreme (Faske et al., 2019; Gray, 2004). More broadly, our predicted effects could be amplified or negated by effects of climate change on life stages not considered in this study (Kingsolver & Buckley, 2020), or by effects on ecological relationships, for example, with host plants or natural enemies (Agosta et al., 2017). Geographical changes in the propensity for range expansion and population outbreaks would be of considerable concern to extensive management efforts to slow spread and protect land from damaging outbreaks (Tobin et al., 2012). However, an important source of uncertainty concerning future projections from this study is that wild *L. dispar* populations can evolve in response to changing climate, possibly enhancing or mitigating the effects observed here depending on the degree to which the thermal performance traits we measured are under selection and the direction of selection in a particular location.

A robust body of literature on insects has shown that warmer temperatures can facilitate range expansion (Lehmann et al., 2020) or can accelerate invasion speed (Seiter & Kingsolver, 2013), but may also negatively impact populations of other species (Haynes et al., 2014; Johnson et al., 2010; Klapwijk et al., 2013). Such variations in thermal responses impede generalization of the response of species to climate change. Our findings, taken together with other studies documenting geographical variation in thermal response of *L. dispar* (Faske et al., 2019; Thompson et al., 2017, 2021), emphasize how the ecological effects of climate change can be spatially heterogeneous, not only due to regional variation in climate change (Karmalkar & Bradley, 2017), but also due to variation in the thermal tolerance and performance of local populations of a given species.

## AUTHOR CONTRIBUTIONS

**Jonathan Walter:** Formal analysis (lead); writing – original draft (lead). **Lily Thompson:** Data curation (lead); investigation (equal); project administration (lead); writing – review and editing (supporting). **Sean Powers:** Investigation (equal); writing – review and editing (equal). **Dylan Parry:** Conceptualization (supporting); funding acquisition (supporting); investigation (equal); methodology (equal); project administration (equal); writing – review and editing (equal). **Salvatore Agosta:** Conceptualization (supporting); methodology (equal); project administration (equal); supervision (equal); writing – review and editing (equal). **Kristine L. Grayson:** Conceptualization (lead); funding acquisition (lead); methodology (equal); project administration (equal); supervision (equal); writing – review and editing (equal).

## CONFLICT OF INTEREST

None.

## Supporting information


Appendix S1

## Data Availability

Data and code accompanying this study are publicly available on Zenodo, https://doi.org/10.5281/zenodo.6583673.
