# Supplementary material for: Growth and development of an invasive forest insect under current and future projected temperature regimes
Source: Ecol Evol. 2022 Jun 17;12(6):e9017. doi: 10.1002/ece3.9017 (PMC9204848; doi:10.1002/ece3.9017)
Supplement: Supplementary file 1 — Appendix S1 [file ECE3-12-e9017-s001.docx]

**Appendix**

**Table A1.** Population information for *Lymantria dispar dispar* including coordinates, elevation, number of generations reared prior to this experiment, and the number of egg masses contributing to the reared population. Egg masses were field collected from relatively low-density populations from across the invasive range at sites where populations were high enough for sampling, but well below outbreak densities. Detection of extremely low populations at the leading edge of an invasion can be prohibitively difficult, thus, some of our populations were sourced from collections made in previous years and reared annually under ambient outdoor conditions. These populations were used when we were unable to find new eggs masses at desired localities. While variation in number of previously reared generations was unavoidable, the pattern among populations is consistent between those that were recently collected and those that were reared for multiple years.

| **Population** | **Region** | **Coordinates**  **(Lat, Long)** | **Elevation**  **(m asl)** | **No. of generations in lab** | **No. of egg masses collected** |
| --- | --- | --- | --- | --- | --- |
| IR | North | 46.6762°N, 91.4469°W | 269 | 1 | 13 |
| AL | North | 46.5967°N, 91.0251°W | 297 | 1 | 21 |
| MN | North | 44.8660°N, 93.2296°W | 261 | 1 | > 30 |
| WI | North | 42.6231°N, 90.5451°W | 243 | 1 | 30 |
| WV1 | South | 38.3747°N, 80.9011°W | 614 | 1 | 60 |
| NC1 | South | 36.4491°N, 76.0246°W | 3 | 7 | 20 |
| NC2 | South | 35.2503°N, 75.5813°W | 1 | 1 – 2* | 11* |

*5 egg masses were freshly collected in 2018; 6 had been reared for 1 previous generation.

**Table A2.** Number of larvae surviving to 5^th^ instar by source population and temperature regime. The experiment began with 25 individuals from each source population in each temperature regime.

|  | **NC2** | **NC1** | **WV** | **WI** | **MN** | **AL** | **IR** |
| --- | --- | --- | --- | --- | --- | --- | --- |
| **Base WI** | 23 | 18 | 25 | 23 | 21 | 24 | 22 |
| **CC4.5 WI** | 24 | 18 | 23 | 23 | 17 | 22 | 23 |
| **CC8.5 WI** | 24 | 23 | 25 | 24 | 24 | 24 | 24 |
| **Base VA** | 21 | 24 | 25 | 23 | 22 | 22 | 24 |
| **CC4.5 VA** | 25 | 24 | 25 | 24 | 21 | 24 | 24 |
| **CC8.5 VA** | 23 | 24 | 24 | 23 | 24 | 23 | 23 |

**Table S3.** Correlations between larval and pupal masses. Correlations are separated by sex due to sexual dimorphism. Data are taken from this study and from Thompson et al. (2021), which used constant temperature thermal regimes.

| **Study** | **Instar** | **Sex** | ***n*** | $\hat{\boldsymbol{\rho}}$ | ***p*** |
| --- | --- | --- | --- | --- | --- |
| This study | 3^rd^ | M | 358 | 0.11 | 0.037 |
| This study | 3^rd^ | F | 272 | 0.07 | 0.075 |
| This study | 5^th^ | M | 354 | 0.30 | <0.001 |
| This study | 5^th^ | F | 266 | 0.37 | <0.001 |
| Thompson et al. (2021) | 3^rd^ | M | 557 | 0.37 | <0.001 |
| Thompson et al. (2021) | 3^rd^ | F | 475 | 0.13 | 0.004 |


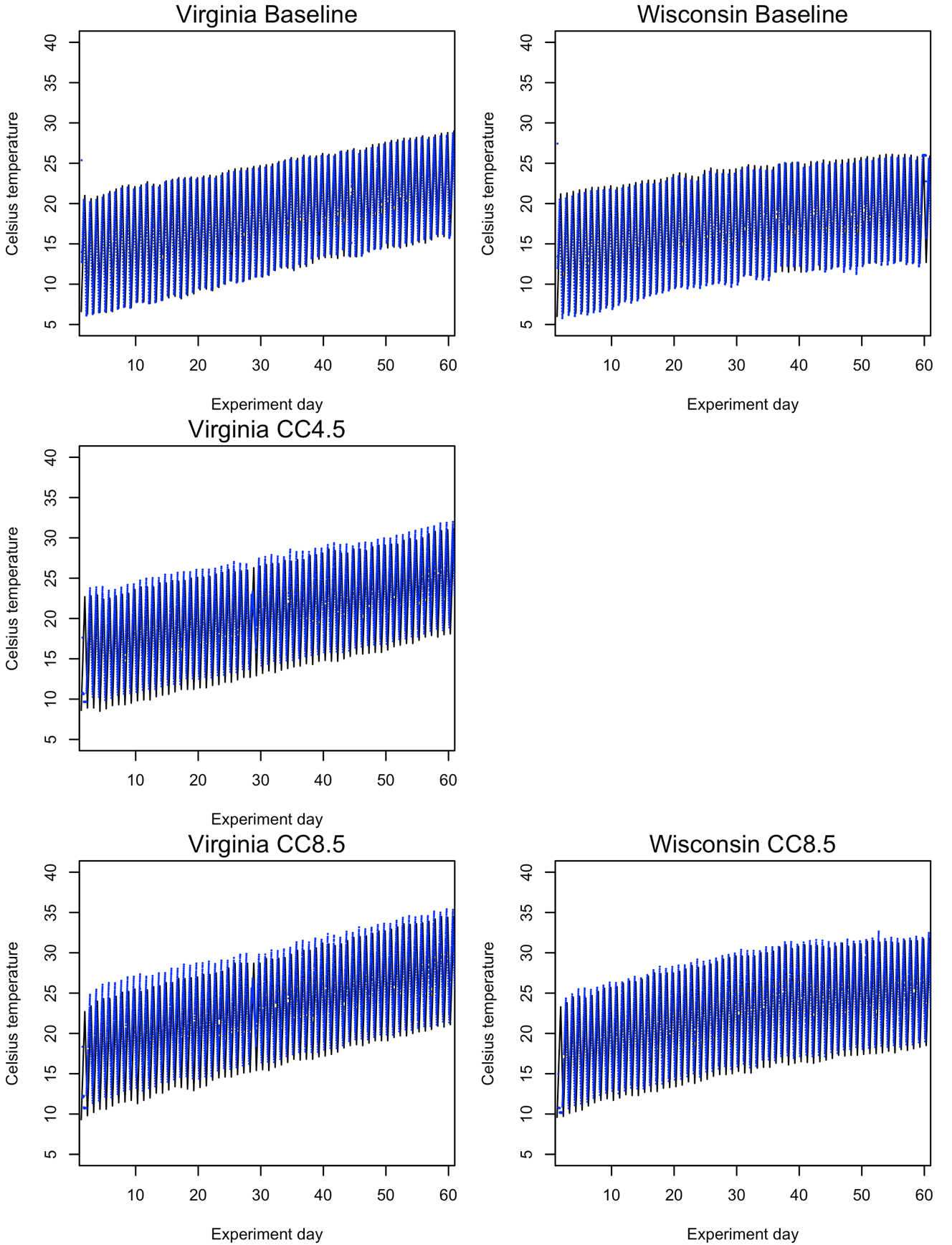


**Figure A1.** Comparison between environmental chamber programmed temperature (black lines) and recorded temperature (blue points). Wisconsin CC4.5 is not shown because the sensor failed to record data. By day 60, every surviving larva had reached 5^th^ instar so the x-axis is limited to days 1 through 60.

**
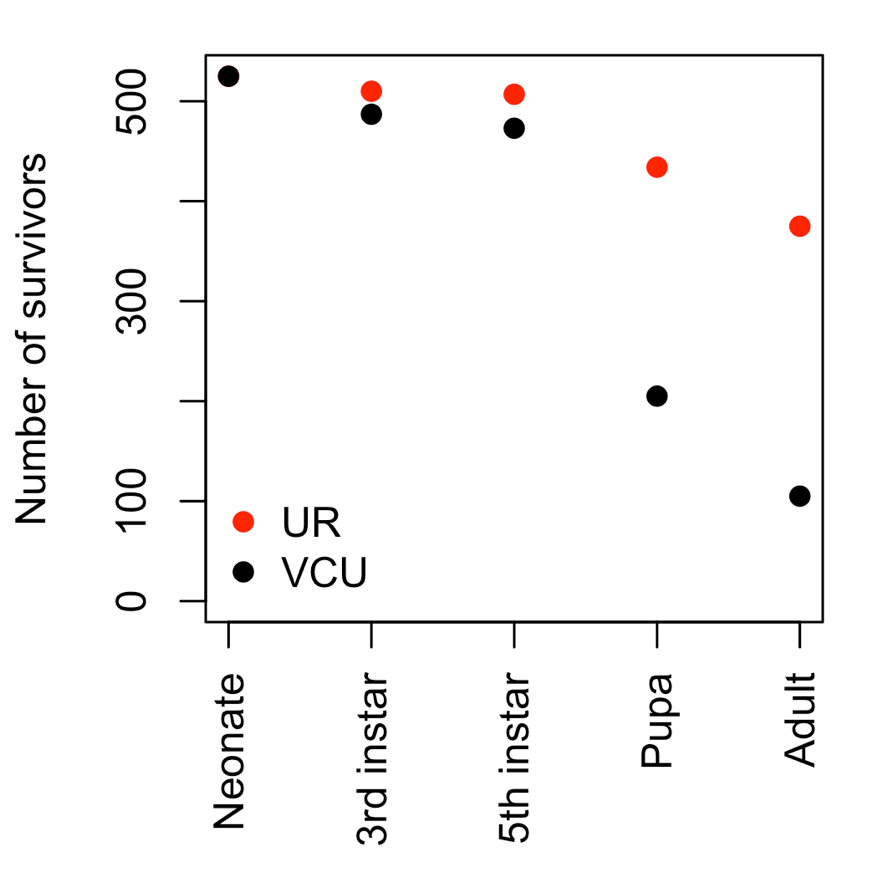
**

**Figure A2.** Number of survivors by life stage and laboratory. Larvae were introduced into environmental chambers as neonates and monitored for development into 3^rd^ and 5^th^ larval instars, pupae, and adults. A mold outbreak in the VCU laboratory caused apparent reductions in survival between 5^th^ instar and pupation, analyses presented in this manuscript focus on data for larvae.


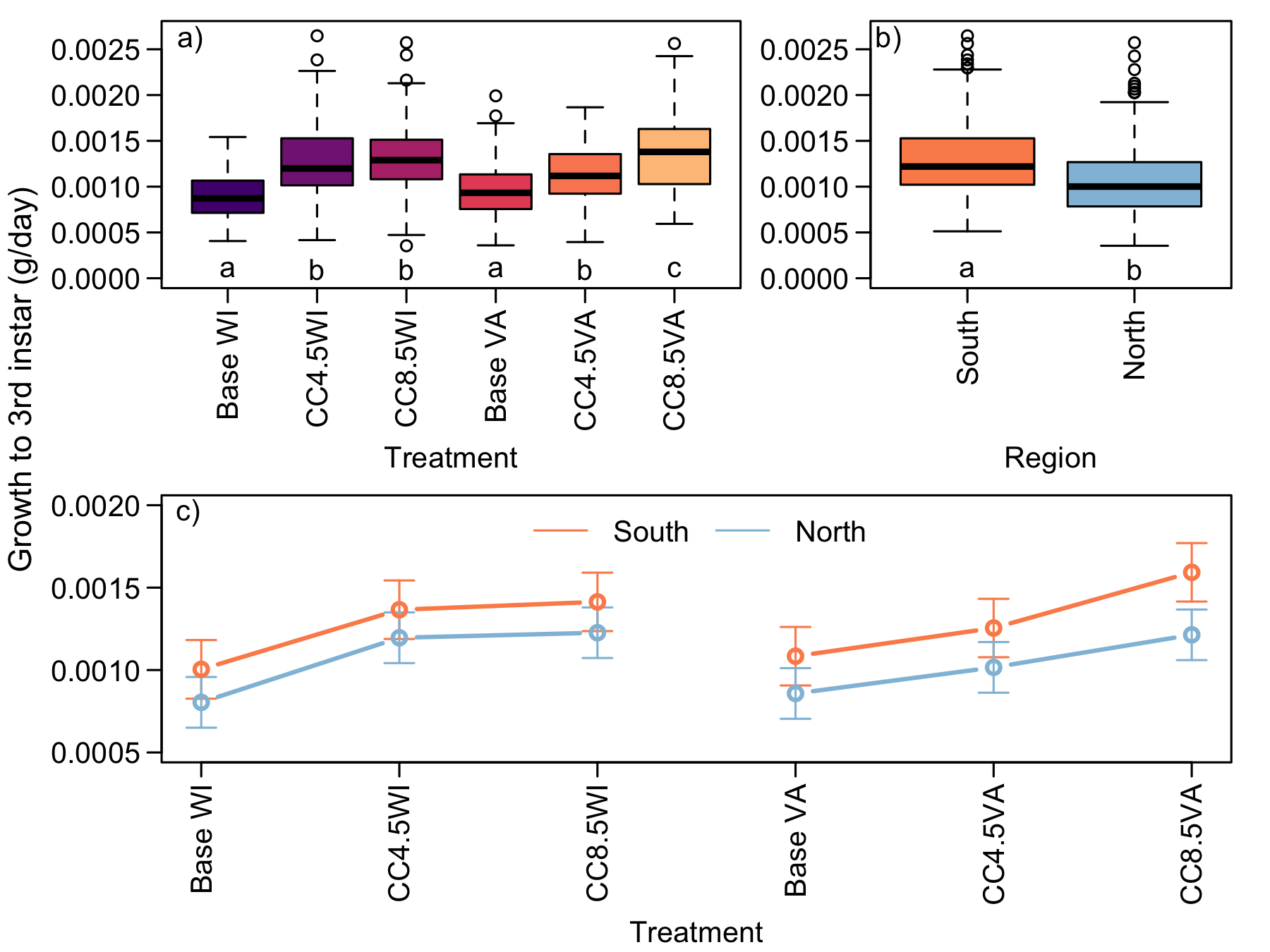


**Figure A3**. Growth rates to third instar (g/day) by a) treatment (*df* = 5, *Χ*^2^ = 339.9, *p* < 0.0001, *η*^2^ = 0.26); b) region (*df* = 1, *Χ*^2^ = 6.61, *p* = 0.010), and c) region×treatment interaction (*df* = 5, *Χ*^2^ = 12.40, *p* = 0.029, *η*^2^ = 0.01) for all individuals. Treatments are ordered from coolest to warmest. Populations are ordered from southernmost to northernmost. Lowercase letters in panels a) and b) denote groups whose elements have estimated marginal means with overlapping 95% confidence intervals. Error bars in c) indicate 95% confidence intervals. Sex was a statistically significant effect (*df =* 1, *X*^2^ = 7.83, *p* = 0.005, *η*^2^ < 0.01) but not of primary interest to our study and so not displayed.


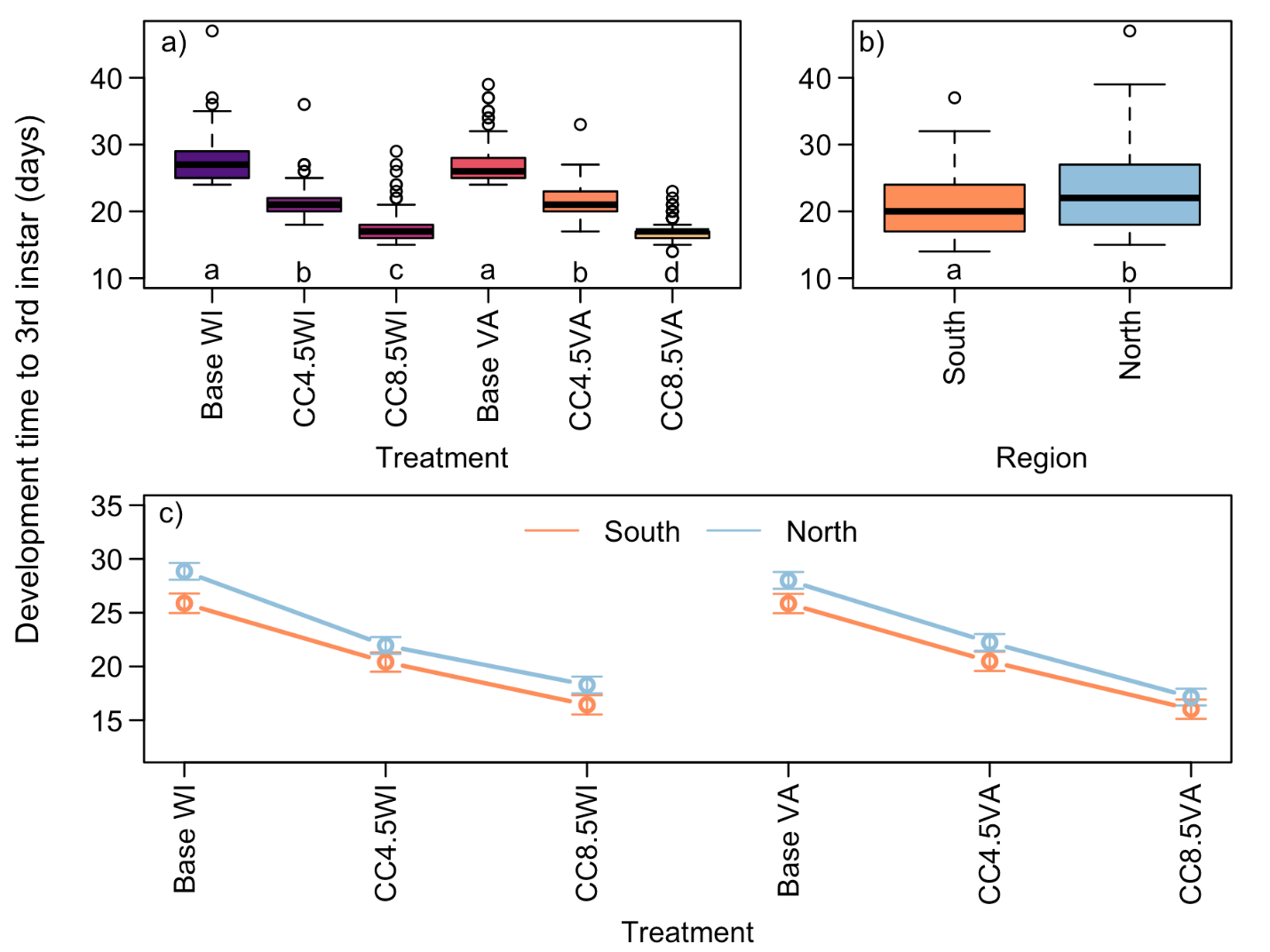


**Figure A4**. Development time to third instar by a) treatment (df = 5, *Χ*^2^ = 3641.5, *p* < 0.0001); b) region (*df* = 1, *Χ*^2^ = 18.34, *p* < 0.0001, *η*^2^ = 0.79); and c) region×treatment interaction (df = 5, *Χ*^2^ = 16.76, *p* = 0.005, *η*^2^ = 0.02) for all individuals. Treatments are ordered from coolest to warmest. Populations are ordered from southernmost to northernmost. Lowercase letters in panels a) and b) denote groups whose elements have estimated marginal means with overlapping 95% confidence intervals. Error bars in c) indicate 95% confidence intervals. Sex was a statistically significant effect (*df =* 1, *X*^2^ = 27.11, *p* < 0.0001, *η*^2^ = 0.03) but not of primary interest to our study and so not displayed.
